# Percutaneous Vertebral Body Augmentation: An Updated Review

**DOI:** 10.1155/2014/815286

**Published:** 2014-04-28

**Authors:** Farzad Omidi-Kashani

**Affiliations:** Orthopedic Research Center, Orthopedic Department, Imam Reza Hospital, Mashhad University of Medical Science, Mashhad 913791 3316, Iran

## Abstract

There are many medical conditions like osteoporosis, tumor, or osteonecrosis that weaken the structural strength of the vertebral body and prone it to fracture. Percutaneous vertebral augmentation that is usually applied by polymethylmethacrylate is a relatively safe, effective, and long lasting procedure commonly performed in these situations. In this paper, we updated a review of biomechanics, indications, contraindications, surgical techniques, complications, and overall prognosis of these minimally invasive spinal procedures.

## 1. Introduction

There are many factors throughout the human life that pathologically weaken the structural strength of the vertebrae and put them at the risk of fracture. Undoubtedly, osteoporosis comprises the most common cause of this weakness and fragility [1]. With an aging population, the prevalence of osteoporotic compression fractures (OCFs) is also increasing.

Since osteoporosis is a contraindication for internal fixation, another solution easily applicable to elderly patients should be employed. The idea of strengthening the weakened vertebral body (VB) was initially raised by Galibert et al. in 1987 [2]. They treated an aggressive vertebral hemangioma at C2 by injecting polymethylmethacrylate (PMMA) into the involved bone. This percutaneous procedure caused almost immediate pain relief. From then onwards, vertebral augmentation is commonly used in the clinical treatment of the patients in need. In this updated review, we briefly discussed the clinical indication, contraindications, surgical techniques, efficacy, and complications of the various methods of vertebral augmentation.

## 2. Clinical Indications

Clinically any pathologic process that reduces the strength of the VB can inevitably increase the fracture risk. These underlying factors may systemically weaken the bone throughout the body or act locally. Osteoporosis is the most common systemic disease that may present with pathologic vertebral fracture. Osteoporosis may be primary or secondary. Primary osteoporosis mainly occurs in postmenopausal women, but many OCFs happen in the patients with osteoporosis secondary to long-term steroid consumption such as the patients with cancer, collagen vascular diseases, transplant therapy, severe allergy, or asthma [3]. The majority of osteoporotic compression fractures will heal with conservative treatment. The typical indication for vertebral body augmentation (vertebroplasty: VP or kyphoplasty: KP) in OCF is refractory local back pain that is related to the fractured VB and not responding to standard medical treatment for 4 to 12 weeks [4, 5].

Vertebral body augmentation has also been used successfully in surgical treatment of acute unstable thoracolumbar burst fractures in otherwise healthy adults [6]. Perfect reduction can be achieved and maintained by careful prone positioning of the patient, short segment pedicular screw fixation, and transpedicular balloon kyphoplasty with calcium phosphate bone cement. This 360° stabilization leads to low rate of implant failure and loss of correction.

**Figure 1:**
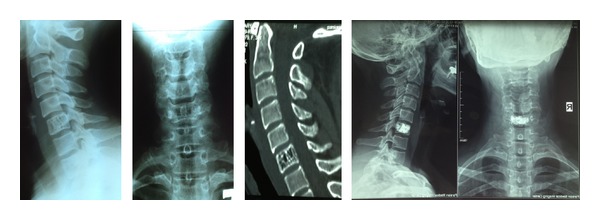
A 32-year-old woman presented with chronic unremitting neck pain. Aggressive hemangioma of C6 vertebra was verified on imaging scans. She was treated with anterior percutaneous vertebroplasty.

Neoplastic lesions comprise one of the other appropriate applications of cement augmentation. The most common neoplastic osteolytic lesions that may present with impending or pathologic fracture and respond well to vertebral augmentation comprise myeloma, metastatic carcinoma (breast cancer, lung cancer, renal cell carcinoma, thyroid cell carcinoma, etc.), benign aggressive tumors like aggressive hemangioma (Figure 1), and etcetera. If underlying pathology is in doubt, bone biopsy can be carried out prior to or accompany the vertebroplasty. The third and last indication of vertebral augmentation is a painful vertebral fracture associated with osteonecrosis [7].

## 3. Who Benefits More from Vertebral Augmentation? 

Undoubtedly, vertebral augmentation is not the first step in the treatment of OCF. The patient who presented with acute OCF (<5 days) without neurologic deficit but is associated with correlating clinical signs and symptoms should be treated conservatively (calcitonin for 4 weeks) [8]. Current literature is unable to strongly recommend for or against bed rest, the use of opioids/analgesics, brace, exercise program (supervised or unsupervised), or electrical stimulation for these cases [9–11]. Medical treatment with ibandronate and strontium ranelate is an option to prevent additional symptomatic vertebral fracture [12, 13].

According to Nieuwenhuijse, the appropriate time for vertebral augmentation in symptomatic OCFs is between two and twelve months after the onset of complaints [14]. In performing vertebral augmentation, it is important to determine that the affected vertebra is the main culprit of the story. Evidence to support this includes local vertebral pain aroused by tapping, high signal intensity on fat suppression magnetic resonance imaging (MRI) scan, and increased uptake in osteoscintigraphy [15]. Increased uptake in osteoscintigraphy is usually observed for two years after the fracture occurs. Due to the difficulty in evaluating images and the considerable costs, it has been recommended that this modality is better to be taken only in lesions that are difficult to identify with MRI [16]. Among these positive predicting factors to VP, concordance of the clinical (localized pain) and imaging findings (bone marrow edema) is the most important [17]. Additionally, a patient with persistent and severe focal back pain related to less than 4 OCFs benefits more from these procedures [18].

## 4. Contraindications

Vertebral augmentation procedures require needling in a prone posture for about one hour on average. Any patient with acute OCF, improvement of symptoms with conservative treatment, asymptomatic VB fracture, tumor mass with spinal canal involvement, presence of osteoblastic metastasis, pregnancy, concomitant uncorrectable coagulopathy, severe cardiorespiratory disease, cement allergy, flexion-distraction or fracture-dislocation injury, and systemic and especially local infection is not a good surgical candidate [19–21]. It is understandable that some challenging situations such as pedicle or posterior VB fracture, vertebra plana with severe vertebral collapse (more than one-third of the original VB height), spinal cord compression, or osteosclerosis of VB trabeculae may increase the complications or hamper needling [19]. Usually, due to unknown natural history of PMMA, vertebral augmentation is not routinely recommended in the patients less than 40 years old [18].

## 5. Surgical Technique

### 5.1. Vertebroplasty

Percutaneous vertebroplasty is the injection of PMMA or bone substitute (like calcium phosphate) into the weakened VB bone. After the local, regional, or general anesthesia was inducted, the patient transferred to the prone position. In this position, a very gentle trunk hyperextension force might be so effective in restoring anterior VB height. However, this maneuver in osteoporotic patients should be performed with caution and with a little force. A high quality biplanar fluoroscopy and proper cement opacification are two most important prerequisites for safe and triumphant VP.

**Figure 2:**
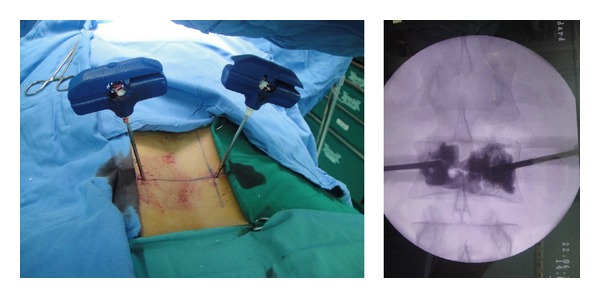
Bipedicular approach in VP. Note the relatively homogenous distribution of the cement through the VB.

**Figure 3:**
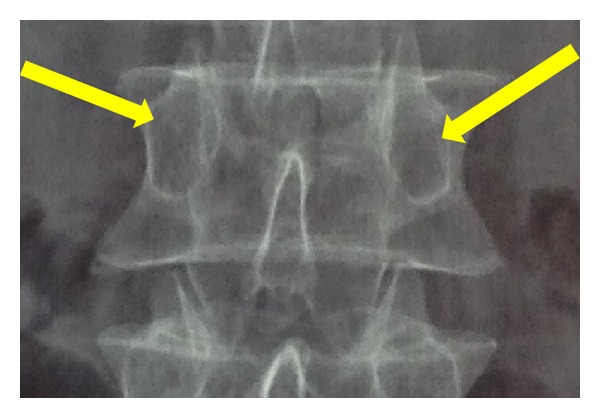
Arrows show the position of the tip of the cannula and also needle trajectory on anteroposterior view, when the cannula came into contact with posterior elements of the vertebra.

Percutaneous needle insertion can be approached anteriorly (in cervical spine) or posteriorly (in thoracic and lumbar spine). Posterior approaches can be applied unilaterally/bilaterally or transpedicularly/extrapedicularly (Figure 2). In posterior approaches for accurate transpedicular needle placement, the surgeon should check the position of the cannular tip relative to the pedicular ring on anteroposterior fluoroscopic projections. When the cannular tip came into contact with the bone of the posterior vertebral element, the tip should be located at 2 and 10 o'clock in left and right pedicular rings, respectively (Figure 3).

When the tip of the cannula passed the junction of middle and anterior third of the VB, 1–4 cc runny cement per side (in bilateral cases) under a relatively high pressure is injected into the weakened VB. This is in contrast with KP that high viscosity cement is injected under less pressure. This difference could explain the higher probability of cement leak in VP versus KP. In VP, high-viscosity PMMA-based cement injection is attempted to be associated with less severe forms of extravasations [22, 23].

On lateral fluoroscopic view, if cement reached the posterior third of the VB, the injection should be stopped to avoid overfilling [24]. It is important to obtain a fairly uniform distribution of cement inside the VB. In some instances this goal could be achieved by only a unilateral approach. Both extrapedicular and transpedicular approaches can be useful in increasing VB strength and stiffness of the involved vertebra but the latter is more capable of restoring VB height due to its easier access to the fracture site [25].

**Figure 4:**
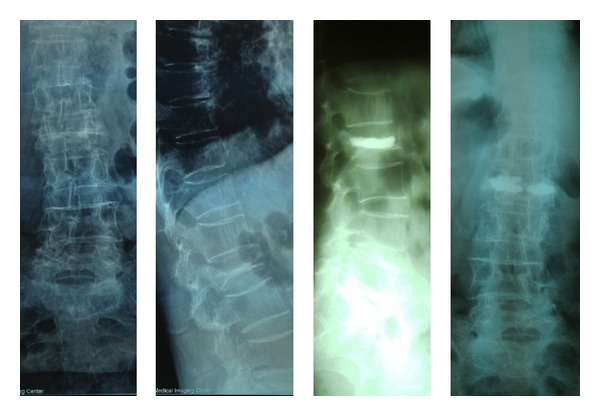
An 80-year-old woman with OCF of L2 (vertebra plana). She was treated with bilateral transpedicular VP.

Literature could not find a strong correlation between the injected cement volume and the amount of VB strength and stiffness restoration, and the degree of clinical improvement [26]. It has been reported that as little as 2 mL cement volume injected into the involved vertebra may restore the initial stiffness [27]. Vertebral augmentation aims to inject the minimum amount of cement required to obtain spinal stability; a good central distribution of cement inside the VB with a vertebral body fraction of 24% was proposed as the optimal fraction to be cemented (Figure 4) [28–30].

### 5.2. Kyphoplasty

Usually KP for thoracolumbar vertebra is carried out by bilateral transpedicular approach. Like VP, initial attempt for vertebral closed reduction is carried out by positioning and traction. Vertebral needling is similar to VP but needles should be replaced with larger cannulae to insert bone tamps through them. In KP, the surgeon aims to centrally place one or two bone tamps inside the VB under biplanar fluoroscopic control.

**Figure 5:**
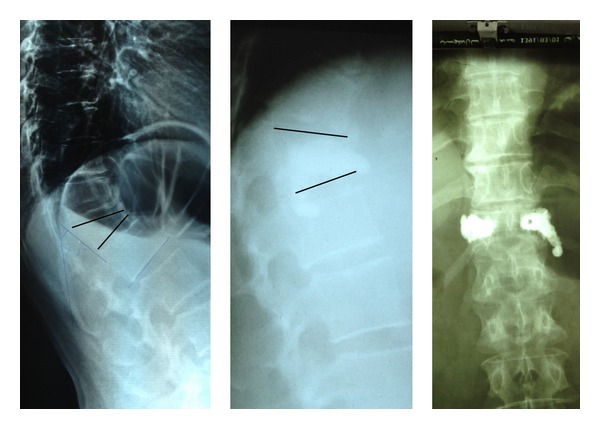
Percutaneous kyphoplasty in a 50-year-old female. Note that cement extravasated into the paravertebral space.

First, bone tamps are inflated under manometric control with radio-contrast medium (for visualization of VB expansion). Usually a balloon pressure of 150 to 300 psi is necessary to reduce OCF. If the procedure is carried out within 3 months of the OCF, usually it is possible to restore 30 to 50% of the primary VB height. Then, balloons are deflated and 3.5 to 8.5 cc of high viscosity cement (with a tooth-paste-like viscosity) under direct image control is injected into the volume previously created by bone tamps (Figure 5) [31]. It should be noted that some authors use a dilator device instead of balloon to expand the collapsed vertebra [32].

## 6. Complications

Like any other surgical procedure, VB augmentations do have some complications. Perhaps, cement leakage constitutes the most common complication of these minimally invasive procedures (27 to 75%) [23, 33, 34]. Fortunately, most of the cement extravasation phenomena are clinically asymptomatic. Cement may leak into the intervertebral disc space (most common), anterior paravertebral area, throughout the needle tract, venous system, intervertebral foramen, or even epidural space (spinal canal) [35–38]. The presence of intravertebral cleft increases the prevalence of complications related to cement extravasation [39]. In the patients with osteolytic tumoral fractures, due to increased possibility of posterior vertebral body fracture, augmentation may be associated with an increased rate of leakage and less predictable pain relief [38]. Central pulmonary cement embolism has also been reported [40]. Factors that have been cited to reduce the possibility of cement leakage during VP include precise needling, sufficient cement visibility, low pressure cement injection, and continuous fluoroscopic monitoring during cement injection [41].

VB augmentation changes the density and loading behavior of the vertebrae and this may cause an increased risk of adjacent vertebral fracture. Many of these osteoporotic patients do fracture more even without any augmentation procedures. Will VB augmentation procedures increase the incidence of subsequent vertebral fracture? It is not proven, currently. Theoretically, excessive filling and augmentation of the VB increase the stress applied to the adjacent osteoporotic vertebra and may cause a following fracture [42]. The majority of the following fractures occur at the adjacent vertebrae and within the first three months of augmentation [43]. It is observed that adjacent vertebral fractures more commonly occur in the patients with previous cement leakage into the disc space. Meanwhile, the effects of supplementary antiosteoporotic drugs on the future fracture risk should not be ignored. Other complications sometimes reported include infection and rib fractures. In KP, rupture of the bone tamp also rarely occurs and usually does not carry any adverse effect.

## 7. Surgical Outcome

It has been verified by numerous studies that VB augmentation procedures in the treatment of chronic OVFs are associated with an immediate, significant, and long acting (>6 months) improvement in back pain and quality of life [16, 44, 45]. In a literature review on efficacy of vertebral augmentation that was carried out by Garfin and Reilley, they also confirmed that both VP and KP have significant effect on pain and function improvement [46]. In comparing VP with KP in the patients with OCF, the results showed that both modalities offer comparable therapeutic effects on pain reduction and disability improvement, although cement leakage prevention and VB height restoration are more pleasant in KP patients [47–49].

In treatment of OCFs, both VP and KP can be effective in restoring anterior VB height [50]. The prone positioning itself has an important role in height restoration. VP also has a safe and beneficial effect on pain and functional status in the patients with spinal tumors or tumor induced VB fractures [51–53].

## 8. Alternatives to Bone Cement in Vertebral Augmentation

Serious complications of cement augmentation including extravasation with its potential neurovascular disastrous effects and uncertain fate of PMMA in the body have led the researchers to consider alternative materials. For example, a transpedicle body augmenter that is a porous titanium spacer has been invented as an internal support to reconstruct the VB [54].

Various materials have been introduced to substitute bone cement in vertebral augmentation procedures. These materials have some degrees of cement properties including good biocompatibility, radio-opacity, and biomechanical strength and stiffness. Some of these materials like composite resin materials, calcium sulfate, or calcium phosphate have passed their experimental stages and are now available clinically [55, 56]. Biomechanically, it has been tested that VB augmentation with calcium phosphate can be effective clinically as well as PMMA in the treatment of OCFs. Due to the fear of uncertain fate of PMMA especially in the young patients, this substance can be an acceptable alternative [57].

## 9. Conclusion

Procedures involving percutaneous vertebral body augmentation are minimally invasive, effective, and long lasting procedures that should be used in properly indicated and selected patients and by experienced and well-educated physicians. Numerous complications are possible but clinically asymptomatic in most patients. Serious neurologic complications are rare but probable; therefore these procedures should be only performed in those well-equipped spinal centers in which emergent neurological decompression is accessible.
